# Disparities by race/ethnicity in unplanned cesarean birth among healthy nulliparas: a secondary analysis of the nuMoM2b dataset

**DOI:** 10.1186/s12884-023-05667-6

**Published:** 2023-05-12

**Authors:** Nicole S. Carlson, Madelyn S. Carlson, Elise N. Erickson, Melinda Higgins, Abby J. Britt, Alexis Dunn Amore

**Affiliations:** 1 Emory University Nell Hodgson Woodruff School of Nursing, 1520 Clifton Road NE, Atlanta, GA 30322 USA; 2 CUNY Graduate School of Public Health & Health Policy, New York, NY USA; 3 University of Arizona, Tucson, AZ USA; 4 Biostatistics and Data Core in the Office of Nursing Research, Emory University Nell Hodgson Woodruff School of Nursing, Atlanta, GA USA

**Keywords:** Pregnancy, Labor, Race, Ethnicity, Racism, Cesarean section

## Abstract

**Background:**

Racial disparities exist in maternal morbidity and mortality, with most of these events occurring in healthy pregnant people. A known driver of these outcomes is unplanned cesarean birth. Less understood is to what extent maternal presenting race/ethnicity is associated with unplanned cesarean birth in healthy laboring people, and if there are differences by race/ethnicity in intrapartum decision-making prior to cesarean birth.

**Methods:**

This secondary analysis of the Nulliparous Pregnancy Outcomes Study: Monitoring Mothers-to-Be (nuMoM2b) dataset involved nulliparas with no significant health complications at pregnancy onset who had a trial of labor at ≥ 37 weeks with a singleton, non-anomalous fetus in cephalic presentation (*N* = 5,095). Logistic regression models were used to examine associations between participant-identified presenting race/ethnicity and unplanned cesarean birth. Participant-identified presenting race/ethnicity was used to capture the influence of racism on participant’s healthcare experiences.

**Results:**

Unplanned cesarean birth occurred in 19.6% of labors. Rates were significantly higher among Black- (24.1%) and Hispanic- (24.7%) compared to white-presenting participants (17.4%). In adjusted models, white participants had 0.57 (97.5% CI [0.45–0.73], *p* < 0.001) lower odds of unplanned cesarean birth compared to Black-presenting participants, while Hispanic-presenting had similar odds as Black-presenting people. The primary indication for cesarean birth among Black- and Hispanic- compared to white-presenting people was non-reassuring fetal heart rate in the setting of spontaneous labor onset.

**Conclusions:**

Among healthy nulliparas with a trial of labor, white-presenting compared to Black or Hispanic-presenting race/ethnicity was associated with decreased odds of unplanned cesarean birth, even after adjustment for pertinent clinical factors. Future research and interventions should consider how healthcare providers’ perception of maternal race/ethnicity may bias care decisions, leading to increased use of surgical birth in low-risk laboring people and racial disparities in birth outcomes.

**Supplementary Information:**

The online version contains supplementary material available at 10.1186/s12884-023-05667-6.

## Introduction

The United States spends more on maternity care than any other high-income country yet has rising rates of severe maternal morbidity and mortality [1–3]. Currently, the U.S. ranks last among high-income countries in maternal morbidity and mortality rates, with approximately 52,000 pregnant people experiencing severe maternal morbidity and 17.4 pregnant people per 100,000 live births dying every year [4, 5]. Substantial disparities in these outcomes exist. For example, Black pregnant people are 3.2 times more likely to die in pregnancy and childbirth and 1.7 times more likely to experience severe maternal morbidity compared to their white counterparts [4].

Over half of pregnant people who experience severe maternal mortality are low risk, meaning that they have no specific identifiable risk factors prior to labor onset [6]. A principal driver of maternal morbidity and mortality in otherwise low-risk pregnant people is unplanned cesarean birth [7]. When unplanned cesarean birth occurs, it increases the risk for postpartum infection, hemorrhage, and other surgical complications, all of which are associated with higher rates of morbidity or mortality [8].

Clinical factors known to contribute to unplanned cesarean include nulliparity, larger fetal size, malposition of fetus, more advanced maternal age, and higher body mass index (BMI) [8]. However, in several prior studies, researchers showed that self-identification as Black increased odds of unplanned cesarean delivery even after adjusting for clinical factors [9, 10]. Moreover, there is evidence of up to a ten-fold difference in cesarean rates by medical center location that cannot be explained by clinical factors [11]. As a primary contributor to maternal mortality/morbidity, unplanned cesarean is key to efforts improving maternal outcomes. However, much of the variation seen in use of this surgery, including variation by maternal race/ethnicity, appears to be related to system-level factors that have not yet been well-described.

Structural racism, which represents the ‘totality of ways in which multiple systems and institutions interact to assert racist policies, practices, and beliefs about people in a racialized group’ (p.1523) [12] is now recognized as a root cause of not only the social determinants of health, but also of pregnancy inequities in the United States [13]. However, the complexity of structural racism has made it difficult to capture by researchers investigating health inequities [12]. A key aspect of structural racism is the identification of people as belonging to a ‘racialized group,’ meaning that they are identifiable by members of their community as belonging to a particular race or ethnicity. The way that a person presents to others racially or ethnically may differ from their ancestry and/or their racial/ethnic self-identification. Thus, presenting race/ethnicity arguably best captures the influence of structural racism because it determines how people are treated as part of a racialized group. A key aspect of this investigation is our use of presenting race/ethnicity as the primary predictor of pregnancy outcomes.

Further investigation is needed to understand the factors contributing to pregnant people’s risk of unplanned cesarean birth, including the influence a laboring person’s presenting race/ethnicity may have on the intrapartum care preceding cesarean birth. The aim of this retrospective cohort study was therefore to evaluate associations between maternal presenting race/ethnicity and unplanned cesarean birth among nulliparous pregnant people whose labors were included in a large national dataset, and secondarily to compare indications for unplanned cesarean by maternal presenting race/ethnicity.

## Methods

For this secondary analysis, we used the Nulliparous Pregnancy Outcomes Study: Monitoring Mothers-to-Be (nuMoM2b) dataset [14]. This dataset was collected in the nuMoM2b multisite prospective cohort study and contained detailed information on the pregnancies of 9,289 nulliparous people who birthed at 8 clinical centers in the United States between 2010 and 2013. Additional details on the nuMoM2b parent study methodology and other investigations using this dataset are available in previous publications [15–18]. The Emory University Institutional Review Board and the Eunice Kennedy Shriver National Institute of Child Health and Human Development both approved this secondary analysis.

The primary outcome of interest was unplanned cesarean birth, which was defined as cesarean birth occurring in individuals with no medical contraindication for vaginal birth who had some period of labor. The occurrence of unplanned cesarean birth and the primary documented indication for cesarean were determined based on medical record reviews performed by the nuMoM2b team.

Participant-identified presenting race/ethnicity was the exposure of primary interest, determined by participant responses to the survey question, “Earlier I asked you to self-identify your ethnicity and race. Now I will ask how other people identify you and treat you. How do other people usually classify you in this country (the United States)?” Options included White, Black or African American or African descent, Hispanic or Latino, Asian, Native Hawaiian or Other Pacific Islander, American Indian or Alaska Native, or some other group. Presenting race/ethnicity was a multiple response variable, and some nuMoM2b participants indicated that they presented as more than one race to different people. We transformed presenting race/ethnicity into a single variable, using the priority system of: Hispanic, Other, American Indian/Alaska Native, Native Hawaiian/Other Pacific Islander, Asian, Black, white.

We selected a sample of cases in the nuMoM2b dataset describing births of nulliparas who reached term gestation (37 0/7 weeks), had no contraindications for vaginal birth (no genital herpes active, vasa previa, velamentous cord insertion, or history of myomectomy) with singleton pregnancies and cephalic-presenting fetuses. We excluded cases if they included one or more of the following pre-pregnancy maternal health conditions: heart disease, coronary artery disease, cancer, HIV, hyperthyroid, bleeding disorder (including blood clots, congenital bleeding disorder, antiphospholipid syndrome), lupus, collagen vascular diseases, RA, ulcerative colitis, autoimmune diseases, liver or gallbladder disease, seizure disorder, thrombocytopenia, kidney disease, herpes, diabetes, hypertension. Cases were excluded if they involved a fetus with a known congenital or chromosomal abnormality. The rationale for excluding cases with pre-existing maternal or fetal health conditions was to reduce the confounding influence of variance in these factors on the occurrence of unplanned cesarean birth. We also excluded cases of nulliparas with a presenting race/ethnicity other than white-, Black- or Hispanic, as other presenting race/ethnicity categories were not well represented in the dataset. After applying all exclusion criteria, the final sample consisted of 5,095 births.

Analyses for study outcomes incorporated variables describing maternal characteristics known to be associated with unplanned cesarean birth, including maternal BMI, maternal age, mode of labor onset (spontaneous labor without augmentation, spontaneous labor with augmentation, or induced labor), development of hypertensive or diabetic complications during pregnancy, and gestational age at the time of birth. Maternal BMI at the time of labor was calculated using participant’s weight, measured at admission to the hospital for labor, and height, which was measured by the nuMoM2b team between 6 + 0 and 13 + 6-weeks’ gestation. Maternal BMI at time of labor was selected instead of pre-pregnancy BMI because the former more accurately reflected participant’s maternal physiology during labor [19–21]. Once calculated, participant’s BMI was categorized into 5 groups based on the World Health Organization BMI cut off points: normal weight (BMI < 24.99 kg/m2), Overweight (BMI 25.00–29.99 kg/m2), Obese I (BMI 30.00–34.99 kg/m2), Obese II (BMI 35.00–39.99 kg/m2), and Obese III (≥ 40.00 kg/m2). A category for underweight BMI (BMI < 18.5 kg/m2) was also initially created, but was ultimately grouped with BMIs < 24.99 kg/m2 due to sparse cell sizes (< 5) in bivariate tests [22].

To assess multicollinearity between predictors, we measured the generalized variance-inflation factor (GVIF). Multicollinearity exists when independent variables are highly correlated with one or more variables in the model. Highly correlated variables, defined as GVIF > 2.24, negatively affect the reliability of statistical inferences. Therefore, predictor variables with GVIF exceeding 2.24 were excluded from the adjusted model [23].

Descriptive statistics were used to describe the population overall and by mode of birth (unplanned cesarean or vaginal birth). These descriptive statistics included maternal and pregnancy characteristics, as well as postpartum maternal morbidity. A composite variable for postpartum maternal morbidity was created, which included occurrence of hemorrhage, a coagulation event, cardiovascular/cardiomyopathy, infection, urinary tract infection, maternal infection, hospital re-admission in the first 14 days postpartum, or an obstetric anal sphincter injury [16]. Categorical data were assessed by calculating frequencies and percentages of responses, while continuous data were analyzed using the median and quartiles, if non-normally distributed. The Anderson–Darling normality test was applied to all continuous data to determine distribution.

To address the study objectives, adjusted multivariate logistic regressions were run, controlling for demographic characteristics and contextual factors, including BMI at time of labor, maternal age, gestational age, development of pregnancy diabetes or hypertension, and mode of labor onset. In the final models, we assessed BMI as a potential mediator or moderator of the association between participant-identified presenting race/ethnicity and unplanned cesarean birth. Interaction analysis using logistic regression was used to test for moderation [24], and structural equation models were run using the Baron & Kenny approach to determine full or partial mediation [25]. Finally, in the group of cases with unplanned cesarean, we conducted sub-analyses to determine the primary clinical indication for cesarean birth and labor duration, stratified by mode of labor onset. Analyses were conducted using R Statistical Software 4.1.2 and STATA MP (Version 17.0), and statistical significance was assessed at α = 0.05.

## Results

### Description of sample

**Fig. 1:**
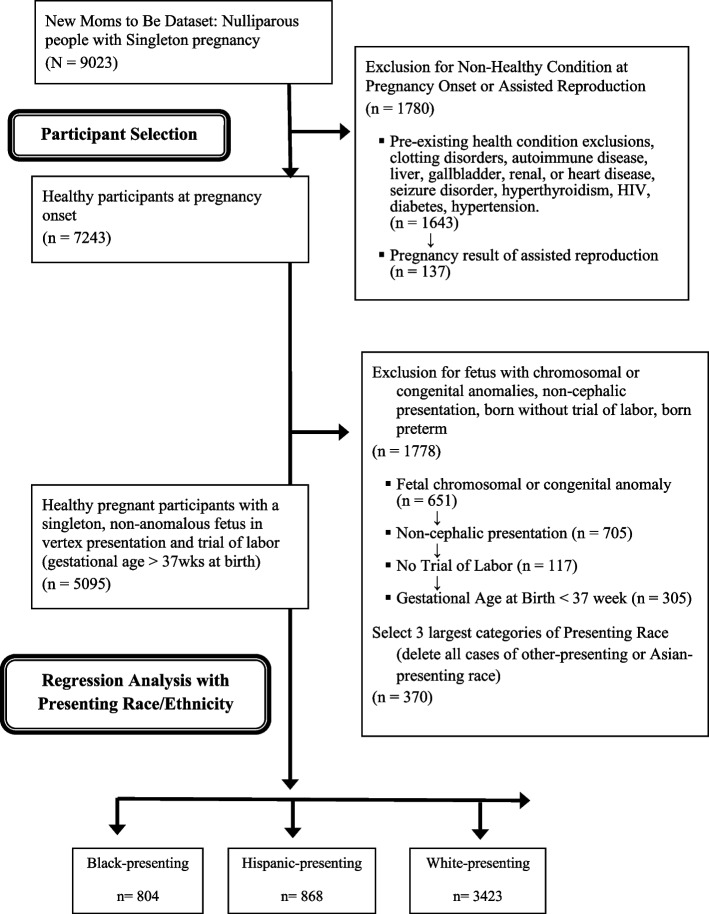
Sample Selection Flowsheet

The majority of individuals in this sample (*N* = 5095) reported being perceived by others in the United States as white (67.2%), while 15.8% were perceived as Black, and 17.0% presented as Hispanic (Fig. 1). Interestingly, some 18.7% -7.2% of participants did not self-identify as the same race/ethnicity they were most frequently perceived as by others in the U.S. (Supplemental Table 1). At the time of hospital admission for labor, 10.9% of individuals in this sample had a BMI in the normal or underweight range, 39.1% were in the overweight range, 27.7% classified as Obese I, 13.0% Obese II, and 9.3% were in the Obese III category. The median maternal age among participants was 27 years (Interquartile range, IQR: 22.0, 31.0) and gestational age was 39.6 weeks (IQR: 39.0, 40.4). Very few nulliparas in this sample developed gestational diabetes during their pregnancy (3.5%), while nearly a quarter developed some form of hypertensive disorder (22.7%).

### Primary outcome: unplanned cesarean birth

Unplanned cesarean birth occurred in 1,001 (19.6%) of the low risk nulliparas in this sample (Table 1). Rates of unplanned cesarean birth were significantly higher among Black-presenting (24.1%) and Hispanic-presenting participants (24.7%) compared to white-presenting participants (17.4%). Cesarean birth also occurred more frequently among those with higher BMI at hospital admission, more advanced gestational age at labor onset, and older maternal age. Unplanned cesarean birth also varied by the mode of labor onset. For example, among the nulliparas in this sample with a cesarean, 53.2% started labor with an induction, compared to only 30.7% of nulliparas ending labor with a vaginal birth. In bivariate logistic regression models, white-presenting participants had significantly lower odds of experiencing an unplanned cesarean compared to Black-presenting participants 0.66 (97.5% CI [0.55–0.79], *p* < 0.001), while Hispanic-presenting participants had similar odds as Black-presenting participants (Table 2).

**Table 1:** Maternal and labor/birth characteristics by mode of birth (*N* = 5095)

	**Total**	**Vaginal Birth**	**Unplanned Cesarean Birth**	***P*-value**^**a**^
n^b^	5095	4094 (80.4)	1001 (19.6)	
**Maternal/Pregnancy Characteristics**				
Presenting Race/Ethnicity				< 0.001
Black-presenting	804 (15.8)	610 (14.9)	194 (19.4)	
Hispanic-presenting	868 (17.0)	653 (16.0)	215 (21.5)	
White-presenting	3423 (67.2)	2831 (69.1)	592 (59.1)	
BMI at Labor Admission (kg/m^2^) n (%)				< 0.001
< 25 kg/m^2^	544 (10.9)	504 (12.6)	40 (4.1)	
25–29.99 kg/m^2^	1947 (39.1)	1690 (42.2)	257 (26.3)	
30–34.99 kg/m^2^	1379 (27.7)	1088 (27.2)	291 (29.8)	
35–39.99 kg/m^2^	648 (13.0)	447 (11.2)	201 (20.6)	
> = 40 kg/m^2^	462 (9.3)	274 (6.8)	188 (19.2)	
BMI at Labor Admission (kg/m2), continuous [IQR]	29.99 [27.01, 34.31]	29.40 [26.59, 33.29]	33.37 [29.19, 38.55]	< 0.001
Gestational Age at Labor Admission, by week, n (%)				< 0.001
37–37.6 weeks	375 (7.4)	311 (7.6)	64 (6.4)	
38–38.6 weeks	774 (15.2)	662 (16.2)	112 (11.2)	
39–39.6 weeks	1570 (30.8)	1311 (32.0)	259 (25.9)	
40–40.6 weeks	1600 (31.4)	1273 (31.1)	327 (32.7)	
41–41.6 weeks	733 (14.4)	509 (12.4)	224 (22.4)	
42 weeks and beyond	43 (0.8)	28 (0.7)	15 (1.5)	
Gestational Age at Labor Admission, median [IQR]	39.60 [39.00, 40.40]	39.50 [39.00, 40.30]	40.10 [39.20, 40.60]	< 0.001
Maternal Age, median [IQR]	27.00 [22.00, 31.00]	27.00 [22.00, 30.00]	28.00 [24.00, 32.00]	< 0.001
Hypertensive Disorder of Pregnancy, n (%)	1154 (22.7)	841 (20.6)	313 (31.3)	< 0.001
Gestational Diabetes, n (%)	177 (3.5)	124 (3.0)	53 (5.3)	0.001
**Labor/Birth Characteristics**				
Mode of Labor Onset, n (%)				< 0.001
not augmented	913 (17.9)	843 (20.6)	70 (7.0)	
augmented	2390 (46.9)	1992 (48.7)	398 (39.8)	
induced	1790 (35.1)	1257 (30.7)	533 (53.2)	
**Postpartum Characteristics**				
Postpartum Maternal Morbidity^c^, n (%)	306 (6.0)	187 (4.6)	119 (11.9)	< 0.001

*Abbreviations*: *BMI* Body mass index, *n* number, *IQR* Interquartile range, *SD* Standard deviation, *L&D* Labor and delivery unit, *NICU* Neonatal intensive care unit

^a^*P*-values for mode of birth obtained for continuous data using Mann–Whitney U test (Kolmogorov–Smirnov test for non-normal distributions). Likelihood ration tests performed for categorical-level data comparisons by mode of birth

^b^Missing values for the following variables: maternal BMI at labor admission (115), hypertensive disorder in pregnancy or postpartum (8), mode of labor onset (2)

^c^Postpartum maternal morbidity event includes occurrence of any of the following: hemorrhage (postpartum hemorrhage requiring transfusion, severe postpartum anemia, or hysterectomy), abnormal coagulation event (postpartum pulmonary embolus or deep vein thrombosis), cardiovascular /cardio- myopathy (postpartum cardiomyopathy or cerebral vascular accident), infection (postpartum endometritis, wound infection or dehiscence, pyleonephritis, urinary tract infection, maternal sepsis, or any other maternal postpartum infections within the first 14 postpartum days), hospital readmission in first 14 days postpartum and/or obstetric anal sphincter injury (OASI), defined as experiencing a 3rd or 4th degree perineal laceration

**Table 2:** Maternal presenting race/ethnicity and cesarean birth in healthy, laboring nulliparas (*N* = 5095)

	**Unadjusted Odds Ratio (95% CI)**^**a**^	***P*-value difference in Model**	**Adjusted Odds Ratio (95% CI)**^**b**^	***P*-value difference in Model**
Presenting Race/Ethnicity				
Black-presenting	Ref	–	Ref	–
White-presenting	0.66 (0.55- 0.79)	< 0.001	0.57 (0.45–0.73)	< 0.001
Hispanic-presenting	1.04 (0.83- 1.29)	0.761	1.17 (0.88, 1.55)	0.278
Maternal Age	–	–	1.08 (1.06–1.10)	< 0.001
Gestational Age at Labor Onset				
37—37.6	–	–	0.90 (0.62–1.28)	0.560
38—38.6	–	–	0.96 (0.73–1.27)	0.797
39—39.6	–	–	Ref	–
40—40.6	–	–	1.33 (1.08–1.65)	0.009
41—41.6	–	–	1.93 (1.50–2.48)	< 0.001
> 42.0	–	–	1.37 (0.59–2.92)	0.439
Development of Gestational Diabetes	–	–	1.18 (0.79–1.74)	0.415
Development of Hypertensive Disorder	–	–	1.35 (1.12–1.62)	0.002
Mode of Labor Onset				
Spontaneous onset, Augmented	–	–	2.00 (1.50- 2.71)	< 0.001
Induction of Labor	–	–	3.24 (2.41- 4.43)	< 0.001
Maternal BMI at Labor Admission				
< 25 kg/m^2^	–	–	Ref	
25—29.99 kg/m^2^	–	–	1.48 (1.03–2.19)	0.041
30—34.99 kg/m^2^	–	–	2.52 (1.75–3.73)	< 0.001
35—39.99 kg/m^2^	–	–	3.52 (2.38–5.32)	< 0.001
≥ 40 kg/m^2^	–	–	4.83 (3.22–7.41)	0.002

*Abbreviations*: *CI* Confidence interval, *n* number

^a^Logistic regression analysis, unadjusted

^b^Adjusted for maternal age, development of gestational diabetes or hypertension, gestational age at labor onset (categorical), maternal BMI at hospital labor admission (categorical), mode of labor onset

In the final multivariate model, unplanned cesarean was associated with maternal age, maternal BMI at or above 25.0 kg/m^2^ (reference group: BMI < 25 kg/m^2^), gestational age at birth between 40.0–41.6 (reference group: 39.0–39.6 weeks), development of hypertensive complications during pregnancy, and labor augmentation or induction (reference group: spontaneous labor without augmentation). These predictor variables were assessed for multicollinearity, and none included in the adjusted model exceeded the recommended GVIF (to the power of ½ degrees of freedom (DF) cut-off point 2.24, range: 1.01–1.08). Therefore, no predictor variables were excluded from subsequent models. In the final adjusted models, compared to Black-presenting participants, white-presenting participants had 0.57 (95% CI [0.45–0.73], *p* < 0.001) lower odds of unplanned cesarean birth, while Hispanic-presenting participants had similar odds of cesarean birth (OR 1.17, 95% CI [0.88–1.55], *p* = 0.28) (Table 2).

It is well-documented that BMI is associated with both race/ethnicity and unplanned cesarean birth [19, 26]. Therefore, BMI at time of labor was assessed as a potential moderator or mediator of the association between participant-identified presenting race/ethnicity and unplanned cesarean birth. We saw no evidence of significant two-way interaction between presenting race/ethnicity and BMI overall (as either a categorical or continuous variable) in predicting unplanned cesarean birth. However, in examining different combinations of BMI and presenting race/ethnicity, we found that BMI was a moderator of the odds for unplanned cesarean among Black-presenting women. Relative to the lowest BMI category (< 25.00 kg/m^2^), higher BMI categories carried higher odds for unplanned cesarean birth among Black-presenting, but not white- or Hispanic-presenting race/ethnicity (Supplemental Fig. 1).

In mediation analyses, we found partial mediation of the direct relationship between presenting race/ethnicity and unplanned cesarean birth by maternal BMI at the time of labor. In the final structural equation model, which included all covariates from the main model (maternal age, BMI, gestational age at birth, development of hypertensive complications during pregnancy, and mode of labor onset), BMI explained 24% of the association between presenting race/ethnicity and unplanned cesarean birth (analyses not shown). Mediation analyses assume, in this scenario, that presenting-race influences differences in BMI or body size, which in turn cause cesarean birth; however, the relationship between body mass index and racial identity is complex, and this study was not designed to allow for causality conclusions. Thus, from these analyses was conclude that 1) even after accounting for maternal BMI, presenting race/ethnicity was associated with unplanned cesarean birth and 2) the relationship between higher BMI and likelihood of cesarean birth was noted specifically among Black-presenting participants.

### Indications for unplanned cesarean birth by presenting race/ethnicity

**Fig. 2:**
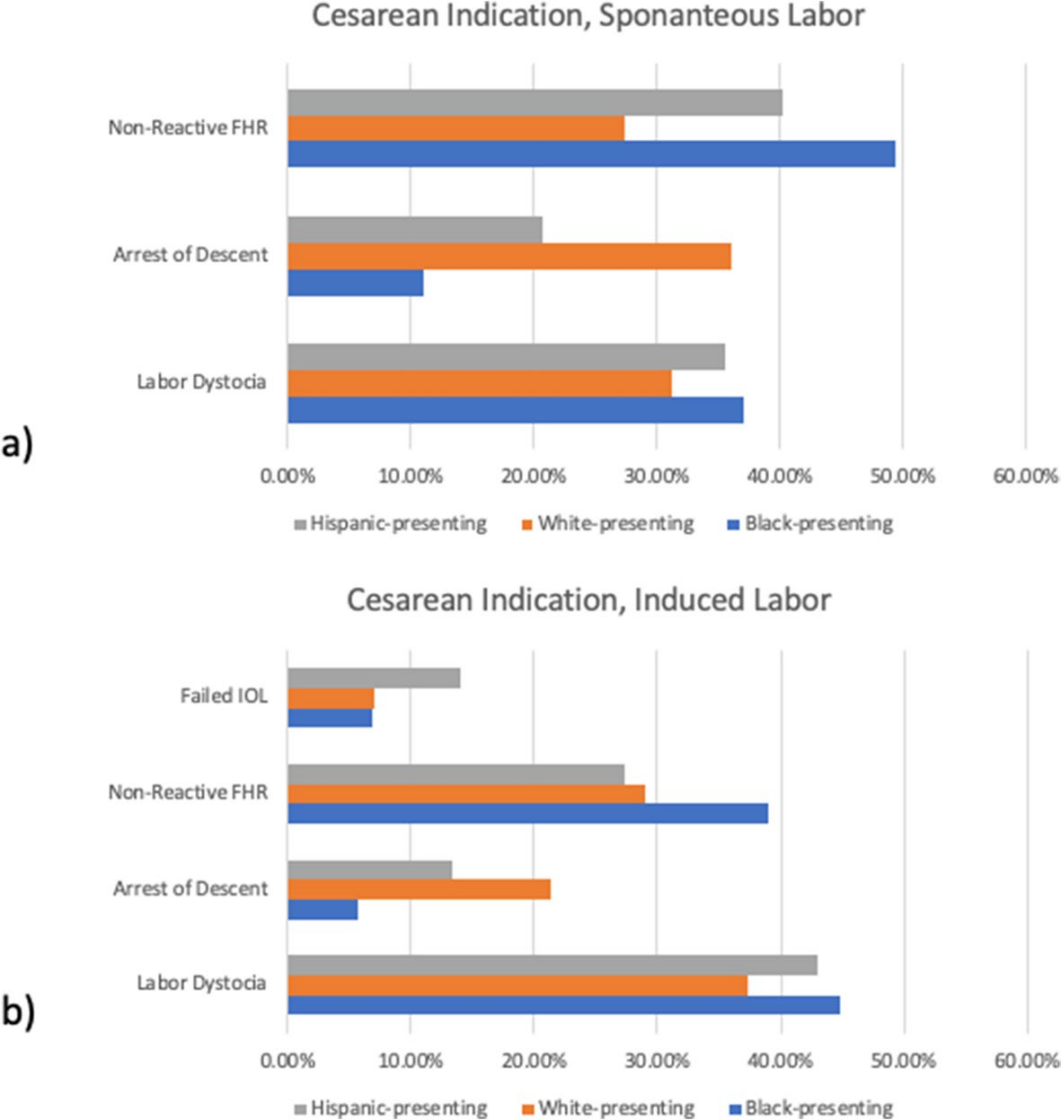
Primary Indication for Unplanned Cesarean Birth by Presenting race/ethnicity and Mode of Labor Onset (*n* = 1001). Abbreviations: FHR, fetal heart rate; IOL, induction of labor. Panel **a** Primary indication of unplanned cesarean in laboring people with spontaneous labor onset (*p* < 0.05 difference between presenting race/ethnicity groups in non-reassuring FHR and arrest of descent), and **b** Induced labor onset (*p* < 0.05 difference between presenting race/ethnicity groups in arrest of descent

Given our findings that the odds of unplanned cesarean birth were different by presenting race/ethnicity, we were interested to see if there were differences by presenting race/ethnicity in the primary clinical indication for cesarean. For this analysis, we focused on a subsample of participants who ended labor with a cesarean (*n* = 1,001). Four conditions identified in the dataset explained nearly all primary indications for unplanned cesarean births: non-reassuring fetal heart rate (FHR), arrest of descent, arrest of dilation, and failed induction of labor (IOL). More Black- and Hispanic-presenting participants who experienced an unplanned cesarean birth were diagnosed with non-reassuring FHR (Black-presenting: 43.80%, Hispanic-presenting: 32.6%) compared to white-presenting participants (28.20%, *P* < 0.001). By contrast, white-presenting participants were more often diagnosed with arrest of descent (28.5%) compared to Black- (8.2%) or Hispanic-presenting nulliparas (16.2%, *P* < 0.001) (Fig. 2).

Among the other primary indications for unplanned cesareans in this sample, we saw no difference by presenting race/ethnicity for either arrest of dilation (labor dystocia) or failed labor induction. To investigate whether intrapartum providers had different tolerances for diagnosing arrest of dilation in nulliparas with different presenting race/ethnicity, we compared duration of labor from hospital admission to birth by presenting race/ethnicity, stratified by mode of labor onset (Table 3). However, we found no evidence of statistical significance between participant-identified presenting race/ethnicity and labor duration, regardless of the mode of labor onset.

**Table 3:** Maternal presenting race/ethnicity and primary indication for unplanned cesarean, stratified by mode of labor onset (*N* = 1001)

	**Spontaneous Labor Onset** (*n* = 468)						**Induced Labor Onset** (*n* = 533)			
**Primary Indication for Cesarean Birth**	n	Black presenting (*n* = 89)	White presenting (*n* = 292)	Hispanic-presenting (*n* = 87)	*P*-value	n	Black presenting (*n* = 105)	White presenting (*n* = 300)	Hispanic-presenting (*n* = 128)	*P*-value
**Arrest of dilation, n (%)**	155	33 (37.1)	91 (31.2)	31 (35.6)	0.5	214	47 (44.8)	112 (37.3)	55 (43.0)	0.4
Time duration from hospital admit to cesarean, median [IQR], hrs	435	13.1 [9.5, 19.1]	13.9 [8.6, 17.3]	13.9 [9.3, 20.4]	0.5	478	21.8 [15.9, 29.5]	20.4 [16.0, 28.1]	22.2 [14.8, 29.2]	0.7
**Non-Reassuring Fetal Heart Rate, n (%)**	159	44 (49.4)	80 (27.4)	35 (40.2)	** < 0.001**	163	41 (39.0)	87 (29.0)	35 (27.3)	0.1
**Arrest of Descent, n (%)**	133	10 (11.2)	105 (36.0)	18 (20.7)	** < 0.001**	87	6 (5.7)	64 (21.3)	17 (13.3)	** < 0.001**
**Failed Induction, n (%)**	0	–	–	–	–	48	9 (8.6)	21 (7.0)	18 (14.1)	0.06

Chi-square test used to test for difference between groups on categorical variables, Kruskal–Wallis test used to compare continuous variables across groups. Missing data: total of 21 cases of people whose labors were induced missing primary indication for unplanned cesarean. Among people who had spontaneous onset of labor, 21 cases were missing primary indication. Missing labor duration for 67/533 people with induction, and 42/468 people with spontaneous labor onset

### Maternal and neonatal outcomes following unplanned cesarean birth

There were significantly worse maternal outcomes following cesarean birth by presenting race/ethnicity, regardless of the indication for the surgery. For example, Black- and Hispanic- presenting nulliparas had roughly double the rates of postpartum maternal morbidity (15.5% and 17.2%, respectively) following unplanned cesarean birth as did white-presenting nulliparas (8.8%, *p* < 0.001, Supplemental Table 2). Compared to white-presenting nulliparas with cesarean birth, Black- and Hispanic- presenting nulliparas had higher rates of SGA neonates (14.9% among Black, 9.8% among Hispanic, and 5.9% among white-presenting people in this sample, *P* = 0.003). However, neonatal outcomes were similar by presenting race/ethnicity; rates of Apgar scores < 7 at 5 min of life and NICU admission were not different by presenting race/ethnicity (Supplemental Table 2).

## Discussion

### Racial disparity in use of unplanned cesarean birth among low-risk nulliparas

In this secondary analysis of a large, multi-site prospective study, we found evidence of a racial disparity in the use of cesarean birth following a trial of labor. Differences were identified among healthy nulliparas, with Black- and Hispanic- presenting nulliparas having roughly double the odds compared to white-presenting nulliparas of ending labor with an unplanned cesarean in analyses adjusting for clinical characteristics. This finding is not new, as several other groups of investigators have seen similar disparities in cesarean birth rates [27–30]. For example, Williams and colleagues recently published evidence of higher cesarean birth rates in Black, Hispanic, and Asian compared to white people in a large retrospective cohort study from two academic medical centers in the same geographic area (*N* = 18,946). They found that Black women’s odds of cesarean were 1.68 (95% CI 1.45–1.96) those of white women in adjusted analyses [27]. Similarly, Stark and colleagues saw higher likelihood of cesarean birth among non-Hispanic Black women compared to non-Hispanic white women in retrospective cohort analysis (*N* = 9,865) of women laboring at a single tertiary care center (OR in analysis adjusting for maternal, perinatal, and systems-level factors 1.58, 95% CI: 1.31–1.91) [29].

We also found evidence that maternal BMI at the time of labor accounted for about a quarter of the influence of maternal presenting race/ethnicity on unplanned cesarean, and that the relationship between BMI and higher odds for unplanned cesarean birth was unique to the Black-presenting race group. In other words, in this sample of healthy nuMoM2b participants it appears that Black-presenting people in larger bodies had the highest risks for unplanned cesarean—higher than white- or Hispanic-presenting people in similar sized bodies. It is possible that there were differences by presenting race and body size during intrapartum care processes that placed this group of people at particular risk for unplanned cesarean birth. This and other possible mechanisms for this finding should be addressed in future research.

### Differences by presenting race/ethnicity in the primary indication for unplanned cesarean birth

This study also shows evidence that non-reassuring FHR was the primary indication for more cesarean births in Black- and Hispanic-presenting, compared to white-presenting people. This finding has also been noted by other investigators [27–31]. In recently published studies, Williams and colleagues found that Black women had roughly double the rates of non-reassuring FHR than white women (37% of cesareans in white women, compared to 64% of Black women and 42% of Hispanic women, *p* < 0.001) [27]. Similar findings were also seen in older studies by several other teams [28, 30, 31]. In a 2022 retrospective cohort study (*N* = 16,687 women), Okinwandu and colleagues found that Black women were 50% more likely to have a cesarean for fetal intolerance (aOR = 1.51, 95% CI: 1.10–2.07) [32]. Those authors theorized that racial differences may be attributed to factors that influence the shared decision-making process for Black women—some of which may be rooted in fear.

Interestingly, we found that fewer cesareans occurred among Black or Hispanic- compared to white-presenting individuals for the primary indication of arrested descent. This means that the higher odds for unplanned cesarean birth we see in Black- and Hispanic-presenting people compared to white-presenting people in the overall sample are even more compelling, as people of color were relatively *less likely* than white-presenting nulliparas to experience unplanned cesarean for arrested descent. This finding raises the question of whether fewer Black-presenting individuals reached the 2^nd^ stage of labor, when arrest of descent was diagnosed. However, nuMoM2b original data abstraction limits us in testing this consideration. We can presume that some of the cesareans for non-reassuring FHR may have occurred during 2^nd^ stage despite adequate progress in descent. It is also possible that the smaller size of newborns from Black- and Hispanic-presenting compared to white-presenting nulliparas contributed to easier second stage descent.

### Differences by mode of labor onset in unplanned cesarean birth differences by presenting race/ethnicity

In this analysis, mode of labor onset was an important predictor of racial disparities in non-reassuring FHR cesarean birth. While racial disparities existed in this outcome among nulliparas who began laboring spontaneously, they were not evident among those with labor induction. Given that induced labor involves use of medications like prostaglandins and higher-duration synthetic oxytocin, which are associated with increased incidence of fetal intolerance [33], it is surprising that racial disparities in non-reassuring FHR cesareans were not found among those with induced labor. To our knowledge, this is the first time that this link between non-reassuring FHR cesarean racial disparity and spontaneous labor onset has been published.

There are two possible explanations for the observation of increased odds of unplanned cesarean for the indication of non-reassuring FHR in Black- and Hispanic- compared to white-presenting nulliparas. First, it is possible that the fetus in nulliparas of color from this sample did not tolerate uterine contractions during labor as well as the fetus in white-presenting nulliparas. Second, there could be differences in the quality of intrapartum care received by Black- and Hispanic- compared to white-presenting nulliparas which predispose them to end labor with a cesarean birth for fetal intolerance. Each of these options is considered in the following sections.

### Racial/ethnic disparities in fetal intolerance of labor

Fetal intolerance of labor has been linked to placental insufficiency secondary to various maternal and fetal factors [34, 35]. However, in sub-analyses of cases from this study with unplanned cesarean birth (Supplemental Tables 2 and 3), we saw few differences in maternal/fetal factors by presenting maternal race/ethnicity. Although maternal age and gestational age were different by race/ethnicity in this group, neither of these were different in Black- presenting compared to white-presenting nulliparas in a direction expected to predict poor outcomes.

There were differences by presenting race/ethnicity in newborn weight for nulliparas ending labor with non-reassuring FHR after spontaneous labor. Smaller infants may be influenced by compromised placental perfusion, which may have many consequences for the birth process [34, 35]. However, there were no differences by presenting race/ethnicity in neonatal Apgar scores < 7 at 5 min or in neonatal intensive care unit admission in the first 28 days of life. Greater antenatal depression has been linked to racial disparities in lower newborn weight; [36–38] however, we did not see differences in pregnancy depression diagnosis by presenting race/ethnicity among those with non-reassuring FHR cesarean and spontaneous labor (analyses not shown).

It is possible that the stress of laboring in an environment where structural racism and/or race/ethnic discordance with the intrapartum care team may place strain on the maternal/fetal unit such that non-reassuring FHR is more likely. In previous investigations of perinatal outcomes among Black presenting populations, experiences of discrimination, racism and general stress were found to increase the risk for adverse perinatal outcomes, such as preterm delivery and low birth weight [36–38]. The nuMoM2b study collected the Experience of Discrimination scale from each participant at their 2^nd^ prenatal visit [14], but these scores were not significantly associated with unplanned cesarean birth when added to adjusted models (analyses not shown). Given that most U.S. pregnant people have a choice about the people who will provide their antepartum care, but not their intrapartum care, patient-reported experiences of discrimination affecting intrapartum outcomes should ideally be collected specifically referencing the intrapartum timeframe.

### Quality of intrapartum care

A second option to explain racial disparities in non-reassuring FHR cesarean birth in this study is differences in the quality of intrapartum care received by Black- and Hispanic- compared to white-presenting nulliparas. For the most part, it is impossible to address this theory due to limitations of the nuMoM2b dataset. For example, nuMoM2b does not contain information on the use of amnioinfusion, nor on other intrapartum interventions commonly used during a concerning FHR period (maternal position changes, intravenous bolus, etc.) Among nuMoM2b variables that could be used to address this theory, there was no difference by maternal presenting race/ethnicity in the use of synthetic oxytocin augmentation or artificial rupture of membranes following spontaneous labor in this sample.

Non-reassuring FHR diagnoses largely explain the massive increase in U.S. cesarean rates over the past four decades [28, 39]. During this time, widespread use of continuous fetal monitoring during labor was not associated with significant improvement in perinatal death or cerebral palsy rates but did increase rates of cesarean and instrumental vaginal birth. Although intrapartum resuscitation for non-reassuring FHR tracings is effective, reverting approximately 63.7% of category II to category I tracings within 60 min [40], there is little standardization of intrapartum resuscitation across the United States. Possibly, this lack of standardization of intrapartum resuscitation guidelines allows systemic racism and other drivers of poor care to affect outcomes. In a 2017 secondary analysis of a multicenter observational obstetric cohort (*N* = 115,000 births), investigators did not find evidence of racial disparities in the utilization of labor management strategies intended to reduce unplanned cesareans performed for failed induction, labor dystocia, and arrest of descent [30]. However, they did not evaluate differences by race in labor management preceding non-reassuring FHR cesareans. More work is needed to evaluate possible racial disparities in the quality of intrapartum care preceding non-reassuring FHR cesarean birth.

### Strengths and limitations

A key strength of this study is our focus on the healthiest labors. By using a large, multi-site dataset for secondary analyses, we were able to focus on a large sample of healthy, term nulliparas with a trial of labor. In previous investigations, researchers attributed increased use of cesarean for non-reassuring FHR in Black- compared to white women to maternal issues, including obesity, medical/obstetric complications, and fetal macrosomia [28]. However, we found that the racial disparity in non-reassuring FHR cesarean birth existed even when the sample was limited to healthy nulliparas. 

Another strength of this study is our use of maternal presenting race/ethnicity, rather than self-identified or medical record-categorized race, as the key predictor in all analyses. Racism is based on perceived differences by other members of a person’s community; thus, presenting race/ethnicity better captures the influence of person- and system-level racism on intrapartum outcomes in this analysis than self-reported race. Although it is important to consider how differences in individual social determinants of health may worsen health outcomes in some populations of people, distribution of the social determinants of health in the United States is largely defined by race/ethnicity [13]. In this way, racism is the structural determinant of the social determinants, and as such it is a key factor to incorporate in studies such as this one that focus on health inequities.

Like any retrospective cohort study, this investigation was limited to demonstrating associations, not causality conclusions. Although nuMoM2b is a rich dataset for secondary analyses, there were multiple variables not collected by the original study’s team which limited this secondary analysis. For example, nuMoM2b contained no information on amnioinfusion, maternal position changes, or other intrapartum interventions commonly used during periods of non-reassuring FHR. We were unable to characterize the extent to which nulliparas in this dataset received interventions to resuscitate their fetus prior to an unplanned cesarean birth. Although nuMoM2b captured the primary and secondary indications for cesarean births as they were documented in participants’ medical records, it is possible that documented indications were not always the truest reasons in the providers’ minds at the time the decision to proceed with cesarean birth was made. Also, nuMoM2b did not contain information describing the intrapartum care received by participants with enough detail to examine the influence of patient-provider racial congruence or professional training of the provider (for example, obstetrician vs. midwife) on unplanned cesarean birth disparities by presenting race/ethnicity.

Finally, this study is limited by how presenting race was identified in nuMoM2b. The gold standard for presenting race/ethnicity as it relates to possible structural racism in healthcare systems/providers would be to collect this information about patients from their healthcare providers (i.e. to collect the healthcare providers’ perception of each research participants’ race/ethnicity). However, presenting race/ethnicity from the perspective of the research participant is as close as this study could get to this ideal.

More research is needed to better elucidate the mechanisms of racial disparities in pregnancy outcomes, including the diagnosis of non-reassuring FHR during labor. These studies should include prospectively collected information on the use of intrauterine resuscitation for non-reassuring FHR changes. If continuous electronic fetal monitoring is used, more information for these studies should be collected from FHR tracings, including variability and presence of decelerations, as well as categorization over the course of labor.

Maternal presenting race/ethnicity, in addition to other variables reflecting structural racism [12], should be incorporated in future analyses of birth outcomes. These studies should also include measures of inequity and mistreatment during labor/birth from the perspective of the birthing person [41–43], as well as maternal/fetal stress hormone levels. The complexity of the experiences of racism among Black presenting pregnant people are poorly captured in many research investigations. This despite the fact that key manifestations of racism during labor/birth have been identified for incorporation in birth outcome investigations [41, 44, 45]. For example, based on their findings from focus groups with African-American women, Nuru-Jeter and colleagues recommended that birth outcomes research capture measures of women’s childhood experiences of racism, as well as their experiences of racism directed at their children and over their life course—all of which could be ‘sources of stress with potentially serious implications for birth outcomes’[44]. In a 2022 publication, Chambers and colleagues identified several system-level variables described by perinatal clinicians as evidence of racism which could also be measured in birth outcome investigations. These include provision of inequitable care, increased surveillance of Black women and families, and the extent to which structural care issues are present in a system (i.e., history of medical racial experimentation) [45]. There are also several validated questionnaires available for incorporation into birth outcome investigations that measure patient-reported experiences of discrimination [41], respect [42], safety, autonomy, communication and other aspects of obstetric racism during the intrapartum course [43]. Such measures expand the focus of intrapartum research to include not only physical outcomes but also the quality of the birth experience. Finally, although racial/ethnic disparities in birth outcomes persist even when social determinants of health like education [46] and income [47] are optimal, more work is needed to understand if intersectional social determinants of health conditions exist that are protective for birth outcomes, including unplanned cesarean birth for the indication of non-reassuring FHR [16].

## Conclusions

In this secondary analysis of a large U.S. dataset, we see evidence of disparities by presenting race/ethnicity in unplanned cesarean birth. Healthy, laboring people of color more often had cesarean for indication of non-reassuring FHR compared to white-presenting people, particularly during spontaneous labor onset. As we seek new strategies to decrease U.S. disparities in maternal morbidity/mortality by race/ethnicity, it is crucial that birth outcomes research incorporates variables which describe intrapartum care preceding cesarean birth, the patient intrapartum experience, the social determinants of health, and measures of structural racism like presenting race/ethnicity.

## Supplementary Information

**Additional file 1: Supplemental Table 1.** Comparison of Presenting Race/Ethnicity by Self-Identified Race/Ethnicity (*N* = 5095). **Supplemental Table 2.** Low-risk Nulliparous Labor People with Spontaneous Labor Onset and Unplanned Cesarean Birth (*n* = 1001). **Supplemental Table 3.** Low-risk Nulliparous Labor People with Spontaneous Labor Onset and Unplanned Cesarean Birth for Primary Indication of Non-Reassuring Fetal Heart Rate (*n* = 159). **Supplemental Fig. 1.** Predictive Margins of Maternal BMI with Presenting Race for Unplanned Cesarean Birth Among Low-Risk Nulliparous People (*n* = 5095).

## Data Availability

The nuMoM2b Study (Nulliparous Pregnancy Outcomes Study: Monitoring Mothers-to-Be) dataset is available for download from the National Institutes of Health, Eunice Kennedy Shriver National Institute of Child Health and Human Development’s Data and Specimen Hub (DASH), which can be found at https://dash.nichd.nih.gov/.

## Abbreviations

nuMoM2b: The Nulliparous Pregnancy Outcomes Study: Monitoring Mothers-to-Be study
BMI: Body mass index
GVIF: Generalized variance-inflation factor
FHR: Fetal heart rate
IOL: Induction of labor
