# Supplementary material for: Disparities by race/ethnicity in unplanned cesarean birth among healthy nulliparas: a secondary analysis of the nuMoM2b dataset
Source: BMC Pregnancy Childbirth. 2023 May 12;23:342. doi: 10.1186/s12884-023-05667-6 (PMC10176719; doi:10.1186/s12884-023-05667-6)
Supplement: Supplementary file 1 — Additional file 1: Supplemental Table 1. Comparison of Presenting Race/Ethnicity by Self-Identified Race/Ethnicity (N = 5095). Supplemental Table 2. Low-risk Nulliparous Labor People with Spontaneous Labor Onset and Unplanned Cesarean Birth (n = 1001). Supplemental Table 3. Low-risk Nulliparous Labor People with Spontaneous Labor Onset and Unplanned Cesarean Birth for Primary Indication of Non-Reassuring Fetal Heart Rate (n = 159). Supplemental Fig. 1. Predictive Margins of Maternal BMI with Presenting Race for Unplanned Cesarean Birth Among Low-Risk Nulliparous People (n = 5095). [file 12884_2023_5667_MOESM1_ESM.docx]

**Supplemental Table 1.** Comparison of Presenting Race/Ethnicity by Self-Identified Race/Ethnicity (N=5095)

|  | **Self-Identified Race/Ethnicity** | | | | | |
| --- | --- | --- | --- | --- | --- | --- |
| **Presenting Race/Ethnicity^a^** | Total | White, non-Hispanic | Black, non-Hispanic | Hispanic | Asian^b^ | Other^b^ |
|  |  | N (%) | N (%) | N (%) | N (%) | N (%) |
| White, non-Hispanic | 3423 | 3176 (92.8%) | 5 (0.1%) | 164 (4.8%) | 1 (0.03%) | 77 (2.2%) |
| Black, non-Hispanic | 804 | 8 (1%) | 685 (85.2%) | 38 (4.7%) | 1 (0.12%) | 72 (9%) |
| Hispanic | 868 | 61 (7%) | 16 (1.8%) | 706 (81.3%) | 27 (3.1%) | 58 (6.7%) |

^a^Presenting race/ethnicity was the exposure of primary interest, determined by participant responses to the survey question, “Earlier I asked you to self-identify your ethnicity and race. Now I will ask how other people identify you and treat you. How do other people usually classify you in this country (the United States)?” Options included White, Black, African American or African descent, Hispanic or Latino, Asian, Native Hawaiian or Other Pacific Islander, American Indian or Alaska Native, or some other group. Presenting race/ethnicity was a multiple response variable, and some nuMoM2b participants indicated that they presented as more than one race to different people. We transformed presenting race/ethnicity into a single variable, using the priority system of: Hispanic, Other, American Indian/Alaska Native, Native Hawaiian/Other Pacific Islander, Asian, Black, white).

^b^Nulliparas with a presenting race presenting race/ethnicity other than white-, Black- or Hispanic, as other presenting race presenting race/ethnicity categories were not well-represented in the dataset.

**Supplemental Table 2.** Low-risk Nulliparous Labor People with Spontaneous Labor Onset and Unplanned Cesarean Birth (n=1001)

|  | **Total sample** | **Black-Presenting** | **White-Presenting** | **Hispanic-Presenting** | **P-value difference by Presenting Race** |
| --- | --- | --- | --- | --- | --- |
| n | 1001 | 194 | 592 | 215 |  |
| Maternal age in years, mean (SD) | 27.9 (5.7) | 25.2 (5.8) | 29.5 (5.1) | 25.8 (5.7) | **< 0.001** |
| Gestational age at Labor Onset, weeks.days, median [IQR] | 40.1 [39.2-40.6] | 40.0 [39.1-40.6] | 40.2 [39.2-41.0] | 40.0 [39.2-40.6] | 0.14 |
| Development of hypertensive disorder, n(%) | 313 (31.3) | 79 (40.7) | 182 (30.8) | 52 (24.2) | **0.001** |
| Cervical dilatation @ Labor admission, cm,  median [IQR] | 2 [1-3] | 2 [1-3] | 2 [1-3] | 1 [1-3] | 0.07 |
| Use of labor augmentation, n(%) | 398 (39.8) | 79 (40.7) | 245 (41.4) | 74 (34.4) | 0.53 |
| Maternal BMI at labor admission, kg/m^2^,  median [IQR] | 33.4 [29.2-38.6] | 36.8 [31.3-41.6] | 32.6 [28.7-37.5] | 33.1 [29.6-38.1] | **< 0.001** |
| Artificial rupture of membranes, n(%) | 552 (55.1) | 100 (51.5) | 336 (56.8) | 116 (54.0) | 0.21 |
| Clear amniotic fluid, n(%) | 644 (64.3) | 110 (56.7) | 393 (66.4) | 141 (65.6) | **0.009** |
| Neonatal birthweight, grams, mean (SD) | 3489.6 (490.3) | 3356.2 (504.8) | 3542.5 (476.4) | 3450.9 (491.2) | **< 0.001** |
| Neonate with weight at Small for Gestational Age (<10^th^ percentile for gestational age) | 85 (8.5) | 29 (14.9) | 35 (5.9) | 23 (9.8) | **0.003** |
| Neonatal Apgar score <7 at 5 minutes, n(%) | 35 (3.5) | 9 (4.6) | 23 (3.9) | 3 (1.4) | 0.15 |
| Neonatal Intensive Care in first 28 Days of life, n(%) | 168 (16.8) | 38 (19.6) | 95 (16.0) | 35 (16.3) | 0.44 |
| Postpartum maternal morbidity, n (%) | 119 (11.9) | 30 (15.5) | 52 (8.8%) | 37 (17.2) | **0.001** |

**Missing values**: 172 values for maternal age, 24 missing cervical dilatation at hospital admission, 4 missing use of artificial rupture of membranes, 1 missing development of hypertensive disorder, 77 missing fluid color, 2 missing neonatal birthweight, 24 missing in maternal BMI at labor admission, 2 missing from small for gestational age variable, 29 missing neonatal Apgar score at 5 minutes of life, 24 missing from neonatal intensive care.

**Postpartum maternal morbidity event** includes occurrence of any of the following: hemorrhage (postpartum hemorrhage requiring transfusion, severe postpartum anemia, or hysterectomy), abnormal coagulation event (postpartum pulmonary embolus or deep vein thrombosis), cardiovascular /cardio- myopathy (postpartum cardiomyopathy or cerebral vascular accident), infection (postpartum endometritis, wound infection or dehiscence, pyleonephritis, urinary tract infection, maternal sepsis, or any other maternal postpartum infections within the first 14 postpartum days), hospital readmission in first 14 days postpartum and/or obstetric anal sphincter injury (OASI), defined as experiencing a 3rd or 4th degree perineal laceration.

**Supplemental Table 3.** Low-risk Nulliparous Labor People with Spontaneous Labor Onset and Unplanned Cesarean Birth for Primary Indication of Non-Reassuring Fetal Heart Rate (n=159)

|  | **Total sample** | **Black-Presenting** | **White-Presenting** | **Hispanic-Presenting** | **P-value difference by Presenting Race** |
| --- | --- | --- | --- | --- | --- |
| n | 159 | 44 | 80 | 35 |  |
| Maternal age in years, mean (SD) | 26.8 (5.6) | 23.9 (5.7) | 29.0 (5.0) | 24.6 (4.7) | **<0.001** |
| Gestational age at Labor Onset, weeks.days, median [IQR] | 40 [39.1-40.5] | 39.8[39.1-40.3] | 40.2 [39.4-40.6] | 39.5 [37.1-40.2] | **0.01** |
| Development of hypertensive disorder, n(%) | 37 (23.3%) | 14 (31.8%) | 19 (23.8%) | 31 (88.6%) | 0.10 |
| Cervical dilatation @ Labor admission, cm,  median [IQR] | 3 [2-4] | 3 [2-4] | 3 [2-4] | 3 [2.3-4] | 0.31 |
| Use of labor augmentation, n(%) | 127 (79.9) | 35 (79.5) | 64 (80.0) | 28 (80.0) | 0.9 |
| Maternal BMI at labor admission, kg/m^2^,  median [IQR] | 32.2 [28.7-36.6] | 33.2 [30.0-38.7] | 32.1 [28.1-36.4] | 31.4 [28.8-34.7] | 0.13 |
| Artificial rupture of membranes, n(%) | 65 (40.9) | 15 (34.1) | 29 (36.2) | 21 (60.0) | 0.11 |
| Clear amniotic fluid, n(%) | 82 (51.6) | 21 (47.7) | 42 (52.5) | 19 (54.3) | 0.55 |
| Neonatal birthweight, grams, mean (SD) | 3360.2 (442.4) | 3260.8 (421.6) | 3465.6 (426.0) | 3244.3 (458.4) | **0.02** |
| Neonatal Apgar score <7 at 5 minutes, n(%) | 6 (3.8) | 2 (4.5) | 4 (5.0) | 0 | 0.43 |
| Postpartum maternal morbidity, n (%) | 18 (11.3) | 9 (20.5) | 6 (7.5) | 3 (8.6) | 0.08 |

**Missing values**: 26 values for maternal age, 1 missing cervical dilatation at hospital admission, 1 missing use of artificial rupture of membranes, 14 missing fluid color, 2 missing in maternal BMI at labor admission, 5 missing neonatal Apgar score at 5 minutes of life.

**Postpartum maternal morbidity event** includes occurrence of any of the following: hemorrhage (postpartum hemorrhage requiring transfusion, severe postpartum anemia, or hysterectomy), abnormal coagulation event (postpartum pulmonary embolus or deep vein thrombosis), cardiovascular /cardio- myopathy (postpartum cardiomyopathy or cerebral vascular accident), infection (postpartum endometritis, wound infection or dehiscence, pyleonephritis, urinary tract infection, maternal sepsis, or any other maternal postpartum infections within the first 14 postpartum days), hospital readmission in first 14 days postpartum and/or obstetric anal sphincter injury (OASI), defined as experiencing a 3rd or 4th degree perineal laceration.

Supplemental Figure 1. Predictive Margins of Maternal BMI with Presenting Race for Unplanned Cesarean Birth Among Low-Risk Nulliparous People (n=5095)


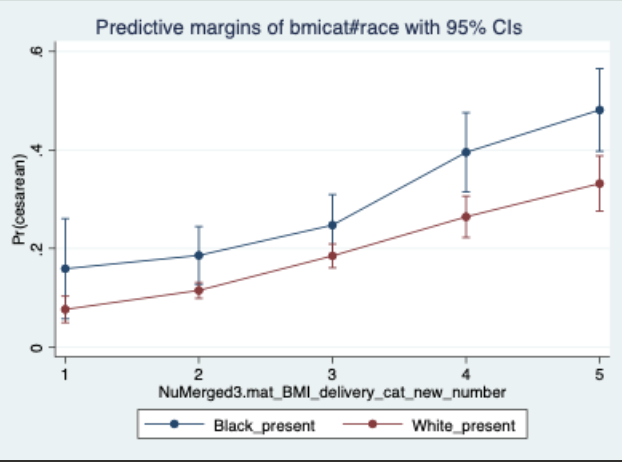


Abbreviations: BMI, body mass index at the time of labor onset; cat, categorical; race, presenting race/ethnicity; cesarean, unplanned cesarean.

Footnote: Margins calculated using logistic regression for unplanned cesarean birth by presenting race, with maternal BMI at the time of labor onset (categorical, 1=BMI <25, 2=BMI 25-29.99, 3=BMI 30.00-34.99, 4= BMI 35.00-39.99, 5= BMI 40.00 or greater), development of gestational diabetes or hypertensive disorder, mode of labor onset, gestational age at labor onset, and maternal age. Shown here: Black- and white-presenting race.
